# Retraction: Icariin reduces dopaminergic neuronal loss and microglia-mediated inflammation *in vivo* and *in vitro*

**DOI:** 10.3389/fnmol.2024.1441744

**Published:** 2024-06-20

**Authors:** Frontiers Editorial Office

Please find the retraction statement below:

Following publication, concerns were raised regarding the scientific validity of the article. An investigation was conducted in accordance with Frontiers' policies. It was found that the complaints were valid and that the article does not meet the standards of editorial and scientific soundness for Frontiers in Molecular Neuroscience; therefore, the article has been retracted.

This retraction was approved by the Chief Editors of Frontiers in Molecular Neuroscience and the Chief Executive Editor of Frontiers. The authors do not agree to this retraction.

